# Immune Functional Assays, From Custom to Standardized Tests for Precision Medicine

**DOI:** 10.3389/fimmu.2018.02367

**Published:** 2018-10-16

**Authors:** Chloé Albert-Vega, Dina M. Tawfik, Sophie Trouillet-Assant, Laurence Vachot, François Mallet, Julien Textoris

**Affiliations:** ^1^Joint Research Unit, Hospice Civils de Lyon, bioMerieux, Centre Hospitalier Lyon Sud, Pierre-Benite, France; ^2^Medical Diagnostic Discovery Department, bioMérieux S.A., Grenoble, France; ^3^EA7426 Pathophysiology of Injury-Induced Immunosuppression, Université Claude Bernard Lyon 1-Hospices Civils de Lyon-bioMérieux, Lyon, France; ^4^Virologie et Pathologie Humaine – VirPath Team, Centre International de Recherche en Infectiologie (CIRI), INSERM U1111, CNRS UMR5308, ENS Lyon, Université Claude Bernard Lyon 1, Université de Lyon, Lyon, France; ^5^Hospices Civils de Lyon, Department of Anaesthesiology and Critical Care Medicine, Groupement Hospitalier Edouard Herriot, Université Claude Bernard Lyon 1, Lyon, France

**Keywords:** immune functional assay, host response, immune monitoring, IGRA, stimulation, critically-ill patients, immunoprofiling, sepsis

## Abstract

The immune response is a dynamic system that maintains the integrity of the body, and more specifically fight against infections. However, an unbalanced host immune response is highlighted in many diseases. Exacerbated responses lead to autoimmune and allergic diseases, whereas, low or inefficient responses favor opportunistic infections and viral reactivations. Conflicting situations may also occur, such as in sepsis where inflammation and compensatory immunosuppression make it difficult to deploy the appropriate drug treatment. Until the current day, assessing the immune profile of patients remains a challenge. This is especially due to the inter-individual variability—a key feature of the immune system—which hinders precise diagnosis, prognosis, and therapeutic stratification. Our incapacity to practically interpret the host response may contribute to a high morbidity and mortality, such as the annual 6 million worldwide deaths in sepsis alone. Therefore, there is a high and increasing demand to assess patient immune function in routine clinical practice, currently met by Immune Functional Assays. Immune Functional Assays (IFA) hold a plethora of potentials that include the precise diagnosis of infections, as well as prediction of secondary and latent infections. Current available products are devoted to indirect pathogen detection such as Mycobacteria tuberculosis interferon gamma release assays (IGRA). In addition, identifying the status and the underlying factors of immune dysfunction (e.g., in septic patients) may guide immune targeted therapies. Tools to monitor and stratify the immune status are currently being studied but they still have many limitations such as technical standardization, biomarkers relevance, systematic interpretation and need to be simplified, in order to set the boundaries of “healthy,” “ill,” and “critically ill” responses. Thus, the design of new tools that give a comprehensive insight into the immune functionality, at the bedside, and in a timely manner represents a leap toward immunoprofiling of patients.

## Introduction

The immune system plays a key role in protecting our body from internal and external threats, contributing to the maintenance of homeostasis. This explains why it is involved is many diseases, being the lead cause, or contributing to their pathophysiology. Excessive or insufficient responses, inborn or acquired, may therefore lead to chronic inflammatory diseases, allergy, or immune deficiencies and increased infectious risk (1). In sepsis, defined as dysregulated host response to infection, the immune system also plays a key role. Our current understanding underline that both pro- and anti-inflammatory responses are involved in a complex and dynamic process, which may lead to organ failures and secondary infections, both contributing to a high morbi-mortality (2). The ability to closely monitor the immune status is thus a critical unmet medical need, which may help stratify patients for personalized care.

However, monitoring the immune system is complex. First, assessing such system relies on a precise knowledge of its components and functions. Innate and adaptive arms of the immune system are both composed of several cell types and humoral components, that act together to maintain immune homeostasis. Counting cells, measuring soluble or cell surface biomarkers, are several options to routinely assess the system (1). However, as many tasks of the immune systems are performed through complex interactions between its components, these routine assays also have limitations and may miss key alterations. Such functional assessment is better performed through Immune Functional Assays (IFA) (3).

Immune Functional Assays are assays that record a response to a given stimulation. Various assays have been developed to better describe or understand the immune system, as well as to monitor diseases in which the immune system is involved. These assays, their advantages and drawbacks, with a special emphasis on their use in sepsis, are the focus of the present review. The development and use of IFA came along with the study of the immune system. Since the times of Edward Jenner, the conception of IFA started when he injected pathogen extracts subcutaneously to assess the humoral response to immunization against smallpox (4). Similarly, Koch and Mantoux noticed that the subcutaneous injection of tuberculin lead to a strong skin reaction in patients with active tuberculosis (TB), and invented the first IFA to diagnose infections (5). Later on, immunologists developed several IFA to assess the immune system and decrypt primary immune deficiencies (PID). Tests such as lymphocyte proliferation or complement assays are still used routinely in the first steps of PID diagnosis (6).

### Immune functional assays to diagnose and manage infections

In tuberculosis, *Mycobacterium tuberculosis* cannot be cultured by classical microbiological techniques so IFA are used to detect a recent contact with the pathogen, and help in the diagnosis of an infection where the causal agent is difficult to isolate and cultivate. Tuberculosis is among the top 10 causes of death worldwide (7). Active TB accounts for 5–10% of the cases and is suspected when the symptoms manifest such as a severe cough that lasts 3 weeks or longer, pain in the chest and coughing up sputum or blood. Active TB is well managed with antibiotics when detected in an early stage. However, latent tuberculosis infection (LTBI, which presents 90–95% of the cases) lurking in the host is asymptomatic and has a high probability of being activated in a hampered immune system. The ability to detect latent TB is therefore critical in situations where patient's management implies iatrogenic immune-suppression such as chemotherapy, transplantation, or chronic inflammatory diseases (8).

Classical tuberculin skin test (TST) has been the only practical mean to diagnose TB over the last century. TST measures the T-lymphocyte response to the intradermal injection of purified protein derivative (PPD) from *Mycobacterium tuberculosis* (Mtb). Positivity to this test is possible from 4 to 12 weeks after the infection and results are obtained in 48 to 72 h after carrying out the test. This test has many limitations as it needs an *in vivo* intradermal injection by trained staff, requires two visits to obtain the results, and its interpretation remains subjective, making assessment difficult. Moreover, its low specificity and sensitivity makes this test barely reliable for active and latent TB diagnosis (8, 9).

In the last few years, T-cells blood based assays have been developed to offer new and more precise diagnostic tools. Interferon-Gamma Release Assays (IGRAs) measure a person's immune reactivity to Mtb (10). IGRA tests have revolutionized the detection of TB as being the first standardized and accurate test currently commercialized. The progress of genomic analysis in mycobacterium including Mtb allowed to find Mtb-specific antigens located into the RD-1 region, early secreted antigenic target (ESAT-6) and culture filtrate protein (CFP-10), which induce strong interferon-gamma (IFN-γ) release from sensitized T cells, signaling an ongoing infection. Since ESAT-6 and CFP-10 are absent from all Bacillus Calmette–Guérin (BCG) substrains (RD-1 region is deleted) and most of non-tuberculous mycobacterium (NTB), these diagnostic tests are not confounded with BCG vaccination and infection with the majority of NTB (11).

**Figure 1:**
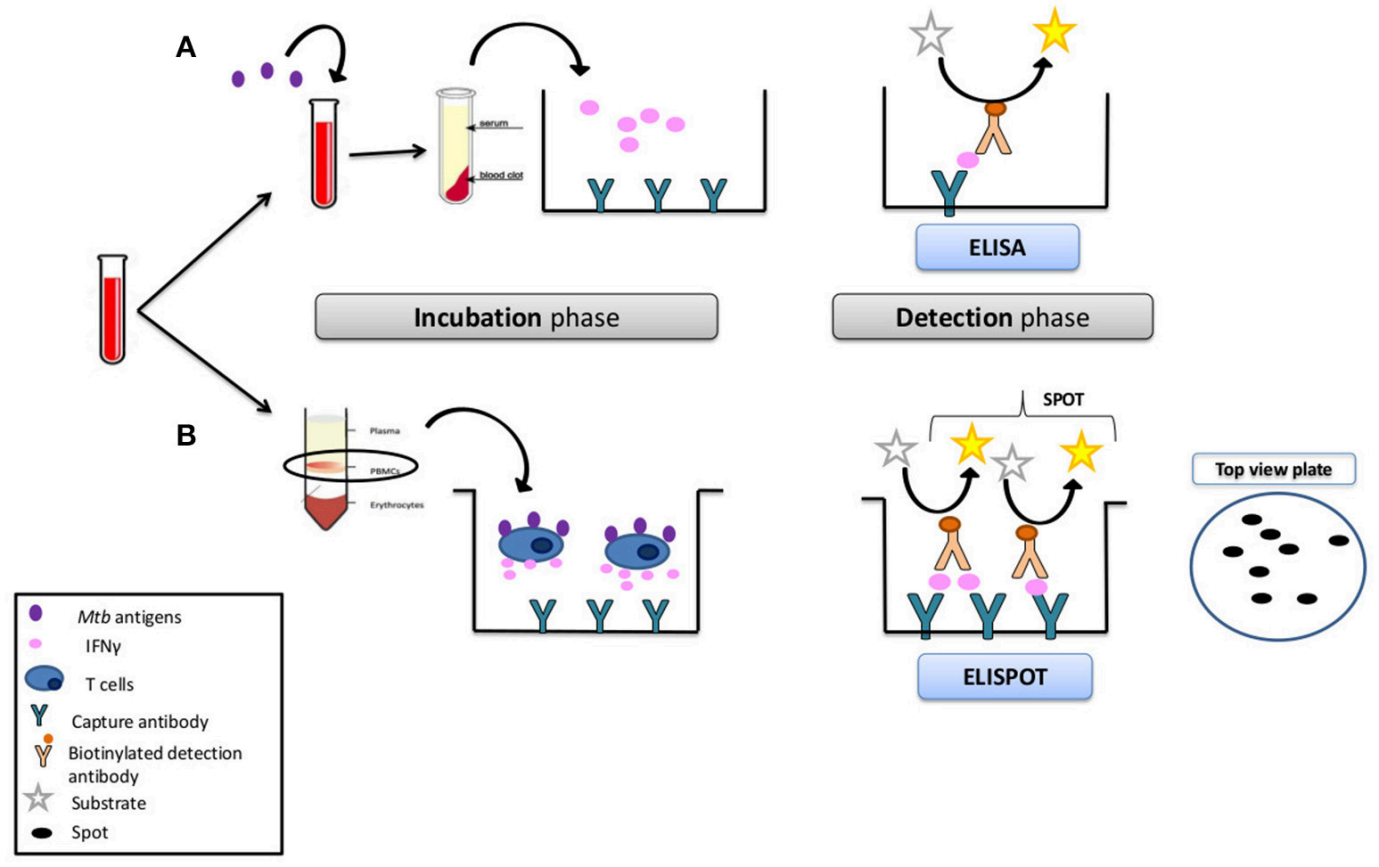
Interferon-gamma-based *in vitro* assays (IGRA). **(A)** Mycobacterium tuberculosis IFN-γ-release assay. In the ELISA method (QuantiFERON®-TB Gold In-Tube Test; Quest Diagnostics, USA), whole blood is stimulated with *M. tuberculosis* antigens, and the amount of IFN-γ secreted into the supernatant is quantified by ELISA. **(B)** In the ELISPOT method (T-SPOT.TB; Oxford Immunotec, UK), PBMCs are prepared by density gradient centrifugation (Ficoll method). A defined number of cells is then stimulated with *M. tuberculosis* antigens for 24 h on plates coated with anti-IFN-γ antibodies. Antigen-responsive cells secrete IFN-γ, which binds to these antibodies. After removal of the cells, antigens are detected by a second labeled anti-IFN-γ antibody. The number of spots on the plate corresponds to the number of IFN-γ+ cells in the sample.

To conduct IGRA test, fresh blood samples are mixed with specific Mtb-antigens and controls, and a specific response is detected through the quantification of IFN-γ release. Currently, there are two FDA-approved commercially available tests: QuantiFERON-TB Gold (QFT) (Qiagen) and T-SPOT.TB (Oxford Immunotec). QFT test uses a peptide cocktail targeting Mtb proteins to stimulate cells in heparinized whole blood. Detection of IFN-γ by enzyme-linked immunosorbent assay (ELISA) is used to identify *in vitro* responses to these Mtb-associated peptides. T-SPOT.TB is a peripheral blood mononuclear cell (PBMC)-based assay which quantifies the number of IFN-γ secreting lymphocytes by ELISpot technique (Figure 1). The advantages of both tests over TST are that they only require a single patient visit to conduct the test, results can be available within 48–72 h. Excellent specificity has been described for latent TB [against controls, (90–91%)], without false-positive in BCG-vaccinated subjects, and a limited number of false-positive due to Non-Tuberculous mycobacterium/Mycobacterium other than Tuberculosis (NTM/MOTT). IGRA test can help determine the full efficacy of BCG vaccine, which can have key implications for its use in current immunization programs as well as in the future development of new improved tuberculosis vaccines (12). Among the disadvantages of TB IGRA is the poor sensitivity to latent TB, poor reproducibility, high number of indeterminate results, high cost, and the inability to discriminate between latent and active TB.

Identification and treatment (i.e., preventive therapy or prophylaxis) of LTBI can substantially reduce the risk of active disease development (by as much as 60%), and is an effective TB control strategy (13). Promising work on new antigens such as mycobacterial Heparin-Binding Haemagglutinin Adhesin (HBHA) antigen might help to improve the ability of IGRA to discriminate between latent and active TB, and identify populations that have dormant TB with a high reactivation potential (14).

Immune functional assays might also be interesting in the management of other infectious diseases. Table 1 lists various pathogens for which IGRA tests have been considered as an option. Human cytomegalovirus (CMV) and Chagas disease are two other examples illustrating how IGRA IFA could help in patient management. In organ transplantation, the risk of CMV reactivation in patients is high and can be well managed with the administration of prophylactic treatment while viral reactivation is easily monitored through specific PCR assays. However, the ability to precisely define the right timing to stop such prophylaxis is pending. Immune Functional Assay could help there by demonstrating patients' recovery and the ability to control CMV replication and fight infection. The main immune response against CMV is cell-mediated, with specific CD8^+^ T cells that produce IFN-γ against CMV. These cells are critical to eliminate viremia from blood. Similarly to TB, IFA tools were developed to quantify IFN-γ and assess the CMV-cell mediated immunity. (e.g., T-Track® CMV kit ELISPOT or QuantiFERON-CMV®). These assays are able to predict risk of developing CMV infection after prophylaxis and can aid in the decision to initiate, delay or discontinue antiviral therapy (15, 16).

**Table 1:** Immune functional assay potentials in identification of latent and/or active infections, monitoring of therapy or vaccination success, and risk stratification for high risk groups.

**Disease**	**Aim of the functional test**	**Target to be explored**	**Action based of the test**
Tuberculosis	Detection of latent infection	Immune memory	Administer prophylaxis
CMV, Epstein-Barr Virus (EBV)	Prognosis of viral reactivation in organ transplant (Risk stratification)	Specific immune competence	Continue or discontinue prophylaxis
Chagas	Monitoring the efficacy of treatment	Immune system activity	Stop treatment
Lyme disease (*Borrelia*)	Diagnosis/ monitoring therapy	Specific immune competence	Adapt therapy accordingly
HIV	Prediction of development of active infection	Specific immune competence	Administer prophylaxis

### Immune functional assays for immune monitoring

Since IFA directly measures *ex-vivo* the capacity of a cell population to respond to an immune challenge, functional testing theoretically represents the best way to monitor immune functions. Although widely used in the research setting, only a few IFA are available routinely in the clinical practice. Most developments have been made along the study of primary immune deficiencies, allergy, and transplantation. However, the rise of immunotherapies in cancer, and the potential applications in sepsis have given these assays a new momentum.

Primary Immune Deficiencies (PID) encompass at least 300 single gene inborn errors, associated to a wide range of phenotypes as diverse as increased risk of infection or malignancies, allergy, or inflammatory/auto-immune diseases (17). The study and characterization of PID has been instrumental in understanding how the immune system works. These studies and the precise diagnosis of PID rely on various IFA that allow the precise characterization of immune defects. Indeed, the clinical and immunological heterogeneity in PID makes diagnosis challenging, while an early and accurate diagnosis facilitates prompt management (18).

Lymphocyte proliferation (also known as lymphocyte transformation test, LTT) is routinely used in clinical immunology labs to assess lymphocyte function. The evaluation of lymphocyte proliferative response is routinely performed by the measurement of tritiated thymidine uptake after stimulation with mitogens or recall antigens (19, 20). For example, children with unusual infections or unusually severe course of infection, that fail to thrive from early infancy (intractable diarrhea, severe eczema), or with recurrent infections with the same type of pathogen should be explored through a protocol that comprise LTT (20). Lymphocyte proliferation assays are also of particular interest when cell counts are normal, such as in Functional T Cell immunodeficiencies (21). However, even if alternatives exist to avoid the use of radioactivity (22), such tests remain cumbersome and difficult to implement.

Chronic Granulomatous disease (CGD) is a relatively rare PID with an incidence of ~1 in 200,000–250,000 individuals characterized by genetic defects in the oxidative burst pathway (NADPH oxidase complexes) that is linked with phagocytosis in myeloid cells, such as neutrophils. Clinically, CGD is characterized by recurrent or persistent bacterial and fungal infections in addition to granuloma formation. Flow cytometric analysis to evaluate NADPH oxidase activity (oxidative burst) are performed using dihydrorhodamine (DHR) 1, 2, 3 as a fluorescent marker of hydrogen peroxide generation before and after stimulation of neutrophils with phorbol myristate acetate (PMA). This is a relatively rapid and highly sensitive assay that allows the use of whole blood without purification of neutrophils, and tends to replace nitroblue tetrazolium test or chemoluminescence tests (23).

The second field where IFA are routinely used in clinical practice is allergy. A typical exacerbated response triggered by food and/or environmental factors is observed in allergy. The gold standard in the field is based on skin prick testing to confirm sensitization in IgE-mediated allergy (24). The recommended method of prick testing includes the appropriate use of specific allergen extracts, positive and negative controls, interpretation of the test after 15–20 min of application, finally a positive result is defined as a wheal of ≥3 mm diameter (25). These tests measure sensitization not the clinical allergy which can be influenced by other factors, interpretation can be liable to over- or under-diagnosis. When there is a confrontation with a doubtful response or limitations to carry out these tests on subjects, the basophil activation test (BAT) is performed. BAT is a flow cytometry based *in vitro* assay that evaluates the expression of activation markers on the surface of basophils after being stimulated with the allergen (26).

The precise monitoring of immunosuppression is also key to ensure long-term viability of solid organ allografts without increasing risk of infection. Monitoring of this dual risks of rejection and infection through immune functional assays could help assess the immune function of the transplant recipient and individualize the immunosuppressive therapy.

Currently, after solid organ transplant or hematopoietic cell transplantation (HCT), levels of immunosuppression are determined by assessing clinical toxicity (e.g., leukopenia, renal failure) and by therapeutic drug monitoring (TDM) when available. However, drug levels are a poor surrogate of the immune status, and may vary a lot among individuals. The main value of TDM is the avoidance of toxic levels. There are currently two IFA available to assess the immune function in transplant patients.

ImmunKnow (Cylex, Inc., Columbia, MD, USA) has been developed to assess the risk of infection and prediction of organ transplant rejection (15). The assay measures intracellular ATP produced by purified CD4^+^ T lymphocytes after *in vitro* whole blood incubation with phytohaemagglutinin (PHA). The samples are incubated for 15-18 h (with or without PHA) and the production of ATP after stimulation is compared to the basal ATP level. Several studies have reported correlation between lower levels of ATP and a higher risk of infection, while increased production of ATP seems to be associated with rejection. The latter could be used as a tool to determine the threshold of immunosuppression and as an indicator of increased risk of infection or rejection (3), such thresholds are hard to determine due to heterogeneity in the studies with various settings and designs.

Pleximmune™ assesses the activity of T-cytotoxic memory cells through the expression of an inflammatory/activation marker: CD154. The expression of CD154 on patient's cells is compared to the basal level of expression on third party cells, an increased ratio being in favor of an acute rejection. This is not an IFA *per se*, as no stimulation step is performed, but rather a surrogate marker of the activity of T-cytotoxic memory cells (27).

In Hematopoietic Stem Cell Transplants (HSCT), recipients exhibit a profound immunosuppression with an increased risk of infection with most pathogens, followed by a gradual recovery. Infections are the most frequent complications after HSCT and have therefore a huge impact on recipient's outcome. Many of these infections are vaccine-preventable infections but no clear vaccination schedule has been established so far and vaccination paradoxically occurs after the highest risk period. In such case, lymphocyte count is not a good indicator as it does not correlate to the vaccine response. IFA may help evaluate the reconstitution of T-lymphocyte pool and immune function recovery after chemo-therapy induced aplasia. Indeed, being able to determine the earliest time for immune responsiveness could dramatically change the prognosis of these patients. The vaccine response in HSCT patients can be measured using conjugate pneumococcal or varicella zoster virus (VZV) vaccine as mitogens (28, 29). In CEREDIH (France) which belongs to the European network RITA (Rare Immunodeficiencies auto-inflammatory and autoimmune diseases network), patients are evaluated for the number of lymphocytes in blood circulation and if those reach a threshold, an *ex vivo* test with PHA is performed. A positive response, i.e., proliferation is observed, allows the patient to be vaccinated. One month after the 3rd booster dose, immune cells are again tested in *ex vivo* condition, this time against recall antigens. A positive response indicates that T cells have recovered and are fully functional.

IFA could be a potential asset in management of sepsis which accounts for 31.5 million cases per year with 6 million deaths and 3 million suffering from frequent hospital re-admission post-sepsis, and long-term morbidity (30, 31). The current definition of sepsis is a life-threatening organ dysfunction caused by a dysregulated host response to infection (2). Although infection is the initial trigger of the response, the dysregulated immune response remains even after the successful treatment of the infection (32). Sepsis patients develop an early hyper-inflammatory response where a cytokine storm is associated to early death and organ dysfunctions. Simultaneously, an anti-inflammatory response tries to compensate causing the patient to plunge in an immunosuppressive phase, thus increasing susceptibility to secondary infections and viral reactivation (33). These responses are very dynamic and vary from one patient to another. The choice of the right treatment can be daunting as the immune status remains hard to predict. IFA could provide useful information on the septic patients' immune status. Being able to assess the immune status at a given point during sepsis time course could be instrumental in reducing morbi-mortality associated to sepsis. What makes IFA superior is the ability to monitor the dynamics of sepsis and possibly stratify patients rather than the static readout in available traditional tests.

The complexity of the sepsis phenotypes are observed not only at the whole-organism level (disease course) but also at the molecular level. It remains unclear which mechanisms drive sepsis-associated pathology and which are secondary disturbances. This is why it is important to understand the biochemical and immunological profile of every patient to help in the dissection of every septic setting (32). A plausible approach is risk stratification to monitor this disease-population. High-risk patients may benefit from earlier clinical interventions, whereas low-risk patients may recover without unnecessary intervention (34).

Researchers have done extensive studies to understand the immune alterations that occur during sepsis providing insights on the valuable biomarkers that can be employed in immunoprofilling of sepsis patients. A range of pro-inflammatory cyto- and chemokines are secreted, and relevant genes are upregulated as an alert state to recruit immune cells, complement, and coagulation systems, endothelial and epithelial cell responses.

Some alterations are observed on the markers expressed on the cell surface such as the decrease of HLA-DR on monocytes, increase of CD64 on neutrophils upon activation, modulation of PD-1 on lymphocytes and other cell markers that can be measured by FACS to help guide patients' management (35). Many of the released proteins can be used as markers to identify the early onset of sepsis and to stratify patients at risk of organ failure caused by the overwhelming inflammatory host-response. Such markers include IL-6, IL-8, procalcitonin (PCT), C-reactive protein (CRP), pentraxin-3 (PTX3), and many others (36, 37).

Nonetheless, in efforts to understand sepsis pathophysiology, it was hypothesized by Boomer et al. that a profound immunosuppression can occur upon sepsis onset and can persist even after the acute hyper-inflammatory phase (38). They conducted a study on the spleen and lungs of septic patients declared clinically dead where they identified changes in the cytokine profile compared to non-septic controls after stimulation with a mitogen. It was observed that both the pro- and anti-inflammatory cytokines production of TNF, IL-6, IL-10, and IFN-γ were impaired at 5 h post-stimulation. Moreover, T-cells expressed higher PD-1, TIM-3, and LAG-3, and a lower expression of CD127 and CD62L, all identified as exhaustion-related markers. The ability to reach levels similar to controls after 22 h of culture in some patients suggest that these alterations might be reversible, and immune recovery possible (38).

The current markers that address the immunocompromised state are the lymphocyte count and human leukocyte antigen- D related (HLA-DR) expression on monocytes measured by flow cytometry (39). Reduced monocyte HLA-DR was found associated to acquisition of nosocomial infections (40), as were elevated regulatory T cells and diminished neutrophil CD88 expression (41). These markers were assessed as stratification tools for immune therapies such as the effect of GM-CSF or rIL-7 on sepsis-induced immunosuppression restoration (42, 43).

A potential gold standard assay to diagnose the immunosuppression is the *ex vivo* stimulation of patients' PBMC with lipopolysaccharide (LPS), measuring TNF-α release with ELISA technique (44). Immunosuppressed patients tend to secrete less TNF-α than healthy subjects, reminiscent of the endotoxin tolerance model, and confirms their immune dysfunction. Limitations reside in the lack of current standardization of such assay, the inter-subject variability in the response to LPS (45), and in the within-individual compartmentalization of tolerance to endotoxin (46).

Neutrophils, key effector cells in clearance of bacteria and fungi infections, can also be assessed by IFA. Indeed, one of the sepsis hallmark is the acquired neutrophil dysfunction that is common during critical illness (47). Phagocytosis, apoptosis, chemotaxis, and oxidative burst are among the neutrophil's defense weapons that are impaired in sepsis setting. Phagocytosis assays (48) are used to assess neutrophils' capacity to clear pathogens; from recognition, engulfment, to intracellular killing. Cells are exposed to *ex vivo* zymosan particles (derived from Saccharomyces cerevisiae cell wall) and incubated for 30–60 min. Phagocytic capacity is determined with light microscopy by the percentage of neutrophils having ingested 2 or more particles (49). Headway to standardize these assays, BD Bioscience-Europe launched PhagotestTM (CE/IVD) which allows the quantitative determination of granulocytes and monocyte phagocytosis in heparinized whole blood. It works with fluorescein (FITC)-labeled opsonized *E. coli* bacteria and determination is performed with flow cytometry. pH sensitive probes also exist in the market (pHrodo® dye) to evaluate neutrophils phagocytosis directly on whole blood, and therefore avoid the variability introduced by sample preparation. Morton et al. tested P4 peptide activity to evaluate severe sepsis patient neutrophils' capacity to engulf and kill bacteria after *ex vivo* stimulation. The increased neutrophil functions observed after incubation with P4 peptide could be determinant in the potential use of augmented passive immunotherapy for patients with severe infection (50). Phagocytosis of neutrophils may be conserved in some sepsis patient while other neutrophil functions may be affected, making these assays insufficient to determine neutrophil impairment. Patients can have adequate phagocytic activity with severely reduced oxidative burst activity, for that, PhagoBurst (BurstTest) from Allele Biotechne, is intended to investigate the altered oxidative burst but also to evaluate the effects of drugs. Cells are incubated with stimuli to promote oxidative burst, and intracellular fluorescence is measured to characterize leukocyte burst activity. Such assays could be of value to stratify septic patients for therapies targeting the innate system such as GM-CSF, and follow-up of the patient's response (51).

Extensive studies were done to monitor the real time changes of the cytokine profile after *ex vivo* stimulation in ICU patients. Antonakas *et al*. compared survivors and non-survivors groups at several time points: 24 h within the first organ dysfunction followed by day 3, 7, and 10. On day 3, PBMCs were stimulated with LPS and defective levels of TNF-α were detected that persisted till day 10. Day 3 recorded the lowest levels in all three cytokines TNF, IL6, and IL8, and they remained low until day 7 compared to the prior time points, this profile was characteristic of the non-survivors' profile (52). A step toward standardization was demonstrated by Monneret *et al*. showing that monocyte anergy could be identified in septic patients by quantifying intra-cellular TNF with flow cytometry following a simple no-wash, no-centrifuge workflow (53).

On the genomic and transcriptomic levels many teams have focused their efforts on identifying signatures that are able to identify immunosuppressed patients at high risk and discover prognosis markers such as the sepsis response signature (SRS1 and SRS2) (54), and the Molecular Diagnosis and Risk Stratification (MARS) consortium (55). In addition, novel bioinformatics approaches, such as a meta-analysis of several studies, showed that it is feasible to develop a prognostic model with good performances (56).

Transcriptome signatures hold great promise to address the complexity of the immune system within a single test, to determine the patient's immune status. Assessing such transcriptional response after *ex-vivo* stimulation may overcome the observed heterogeneity of sepsis cohorts, and provide a better assessment of the immune status on top of several confounders such as inter-individual variability or temporal effects. This approach is now possible with the recent advances in “omics” and multiplexing technologies.

### Current challenges and future perspectives

#### Factors having an impact on immune response

In physiological context, different parameters impact the human immune system. Several studies have highlighted different factors causing inter-individual variability which consequently has an impact on the evaluation of the immune function. Intrinsic factors like age, sex, co-morbidities, heterogeneity of blood composition, and genetic—and even epigenetic—factors are responsible for the physiological variations and differences observed in response to a pathogen challenge among healthy individuals (57–59).

Age represents one of the contributing factors to variability, in particular extreme ages. Two periods of life represented by neonates (especially preterm neonates) and the elderly are often marked by impairments in the immune system. The development of immature immune system in newborns and the deterioration of immune function in the elderly contribute to higher risk of infections observed in these populations. The term immunosenescence has been coined to describe the progressive deterioration of immune system with aging, notably characterized by a decrease of the immune memory. Other flawed mechanisms include, an inverse CD4/CD8 ratio, loss of naïve T cells, increase in the numbers of well-differentiated T cells and alteration of natural killer cells are all hallmarks of immunosenescence (60). However, functional rather than anatomical impairment is the probable underlying cause of immune alteration which can be accompanied by a defective production of inflammatory mediators (61).

The decrease in immune memory related to immunosenescence highlights the key role of this cellular repertoire against infections. Vaccination is one way to induce this immune memory to confer protection against pathogens. It is well known that vaccines act on the adaptive arm of the immunity essentially through the production of antibodies which targets specific pathogens. Recently, new approaches of vaccination target the innate arm of the immunity to protect against various pathogens and clear infections. Arts et al. studied the epigenetic changes, specifically the “reprogramming” of monocytes post-BCG vaccination. Monocytes stimulation with BCG led to functional changes in the innate immunity especially in the pro-inflammatory cytokine profiles. The investigators observed that this intervention led to the clearing of unrelated viral infections such as yellow fever virus (62). BCG vaccination can generate innate immune memory, also known as “trained immunity” that can help prevent certain respiratory infections and neonatal sepsis. To evaluate the efficacy and success of vaccination, recall antigens are used to elicit an *ex vivo* response in order to evaluate whether the induced protection is based on the principle of prior exposure (63). Consequently, the immune response observed after *ex vivo* stimulation in an IFA is largely impacted by innate and adaptive immune memory, contributing to the inter-individual variability.

Besides protection obtained through preventive interventions, diverse studies have highlighted the importance and role of genetic factors in the safeguard against infections. A recent study by Piasecka et al. attempted to delineate the inter-individual variability by exploring the effect of host intrinsic factors such as genes, gender and age on the transcriptional responses and immune cells proportions of healthy volunteers. A thousand donors' blood stratified by age and sex was tested before and after the immune activation with different microbial stimulus that included bacterial, fungal, and viral. The transcriptional response of 560 immune-related genes was quantified as well as the measurement of eight major immune cell types. Finally the investigators measured the contribution of genetic factors to the immune gene expression variation by mapping the expression quantitative trait loci (eQTLs) for associations between genome-wide SNPs and 560 expression traits. The study concluded that the effect of age and gender was moderate but widespread across numerous immune genes and was not relevant to the immune cell composition. Meanwhile, genetic variations had a stronger effect on the regulation of immune genes although they affected only a limited number of gene set, with some genetic variants elucidated their regulatory effect only upon stimulation (64).

Finally, in the recent years light was shed on the important role of the microbiota to alter and modulate the immune response. A conception was developed implicating that symbiosis between immune system and the microbiota can establish a threshold of activation and regulation to maintain homeostasis (65). The disruption of this “alliance” caused by injury or antibiotics was associated to several disorders such as autoimmune diseases, allergy, and even cancer (66). New research observed that critically ill patients, in particular sepsis patients, had a significant shift in the gut microbiota populations marked by the disappearance of bacteria genera that are essential in the host metabolic activity and anti-inflammatory function, which might explain the diversity encountered in the immune status of these patients (67).

Diversity conferred by those intrinsic factors are responsible for the inter-individual differences observed in the immune response and accounts for almost 20% of the variability in the immune response (64). Knowing this inter-individual variability observed in the healthy physiological context, the definition of “Healthy Immune System” has to be well established in order to measure the immune function in a pathological condition.

This concept has been indirectly addressed in the design of IFA in order to be able to interpret patients' results (Table 2). For example, Neuvonen et al. tested the recommended antigens by the WHO for delayed hypersensitivity skin test in a large cohort of healthy population thus setting references for assessing the immune-competence in patients (73). In like manner, Pottumarthy et al. used healthy responses to evaluate the potentials of replacing the traditional skin test with tuberculin gamma interferon assay (74). While, Ulrichs et al. tried to grade the use of ESAT-6 as specific antigen in the development of the IGRA test comparing stimulations of healthy and patients with tuberculosis (75). The Milieu Interieur consortium took the initiative of setting preliminary reference ranges of a healthy immune response and its natural heterogeneity. They challenged healthy blood with different types of stimulus, from Toll-like receptors (TLR) agonists to complex whole microbes, to evaluate and decorticate the response of the immune cells. Healthy population from European ancestry was selected to reduce the inter-individual variability among the donors. Decreasing inter-individual variability readout is a relevant and key aspect to consider when designing an IFA, especially for the interpretation of the results. The efforts of the group highlighted the separation of different immune arms based on induced inflammatory signatures, which can contribute to the monitoring of immune function and the possibility of quantifying dysregulations (76). Setting reference is indispensable for the evaluation of the “in-range” and “out-range,” as a base to map and identify disorders accordingly.

**Table 2:** Immune Functional Assays applications in different pathologies.

**Disease area**	**IFA**	**Assessment**	**Evaluation**	**Reference**
Allergy	Skin test	Diagnosis of food allergy	Wheal diameter on skin	Gupta, (24)
	Basophil Activation test	Diagnosis of food allergy	Markers for basophile granulocytes identification	Hoffmann, (26)
	Histamine Release test	Diagnosis of drug allergy	Histamine release	Dona, (68)
Primary Immunodeficiency Disorder (PID)	Oxidative burst	Diagnosis	NADPH oxidase activity	Abrahams, (23)
	Lymphocyte transformation test	Diagnosis	Proliferation	Moylett EH, (69)
Tuberculosis	IGRA	Diagnosis	IFN-γ release	Van Pinxteren, (70)
Immunosuppression	Endotoxin test	Diagnosis anergy	TNF-α secretion	Cavaillon, (44)
Lymphoma	TLR agonist stimulation on PBMC	Response to therapy	Cytokine profile evaluation	Dietsch (71)
Autoimmunity	IGRA	Biotherapy	IFN-γ release	Mir Viladrich, et al., (72)
Organ Transplant	Immuknow	Outcome prediction	Intracellular ATP production	Lindermann, (3)
Vaccination	Recall antigens for lymphocyte proliferation	Immune function evaluation	Lymphocyte proliferation	Disteler, (28), Hoshina, (29)

#### Need of standardization and precision medicine

Taking into consideration the challenge to interpret results due to inter-individual diversity, technical variability should be kept minimum to avoid complexity of interpretation. IFA is composed of three main parts; the biological sample, the stimulant, and the cellular response, where the aim is to minimize technical variability to increase reproducibility.

Currently, the wider matrix used in IFA are PBMCs which despite technical advances in immunophenotyping, still relies on 50-year-old artisanal skills for the separation of mononuclear cells from whole blood using the Ficoll method (77). Besides, this technique has many limitations as being time-consuming and causing non-specific cell activation and cell death, thus reducing the quality of the sample. Moreover, sample manipulation increases the risk of contamination and introduces technical variability linked to the investigator. It is challenging to standardize protocols within and across laboratories. Some techniques such as intracellular cytokine staining, isolation of specific cells and *ex vivo* stimulations are accompanied with technical complexity and hard access to the testing platform. A primordial requirement of an IFA test is to preserve cell composition and interactions among cell populations and soluble factors should not be disturbed. Hence, whole blood is a more practical solution since it contains the same mix of cells and factors that reflect the inner environment of the subject with minimal handling.

The selection of stimuli to challenge immune cells is of relevant importance to promote a reiterative response at every use. LPS, a specific TLR-4 ligand, is the most widely used compound to stimulate whole blood, PBMCs and most of the isolated cell-population. LPS is purified from gram negative bacteria, nonetheless the difference in bacterial source elicits a distinct response and has different effects on the cells (78). Method of preparation and purity level (79) of LPS adds a higher degree of variability, making its reproducibility difficult from one study to another and the elicited response is hardly comparable. Arens and team illustrated this point when they stimulated *ex vivo* whole blood from controls and sepsis patients with LPS and no statistical difference was observed. Although it is commonly used across laboratories, LPS is not always a good-group discriminator, even between healthy and sick subjects (80). Moreover, the stimulation time is a critical factor in IFA design, since a short boost has an influence on the early and acute gene responders, while a longer period of incubation will favor the stimulation of a long-term response; influencing the arm of the immunity that comes into play.

Unbiased-immune response evaluation after *ex vivo* stimulation is a key part in the development of an IFA. It is widely accepted that only one marker is not enough to diagnose a clinical condition, predict an outcome, or assess a treatment. A combination of biomarkers is favored to obtain a holistic view of the patients' immune status to drive therapeutic decisions. The emergence of -omics studies have led to the development of advanced technologies and ready-to-use devices. Multiplexing in proteomics and transcriptomics allow the analysis of a high number of targeted analytes at the same time. Although caution must be taken with automated algorithms, readout and interpretation must be customized according to the clinical context. As one size does not fit all, in case of sepsis the interpretation must take into account intrinsic factors like SNPs or the natural diversity of patients, external factors such as pathogen (including the site of infection, load, and virulence) which in turn might alter the readout due to the pleiotropic nature of the immune response. A criteria was recently proposed by Shanker-Hari et al. to employ biomarkers and genetic signatures in precision medicine to address response and host heterogeneity. The team suggested that the patient endotypes should be consistent that is, when a pattern is observed among a population, it shall remain the same and persevere against bootstrap testing. The second aspect is to stratify the patients into subgroups, and each subgroup should be biologically and clinically plausible based on the systematic collection of a relevant and known biological basis. Finally, the patient groups should be realistic and are feasible to be clinically managed (81). This approach is already being applied in many research, where sepsis patients are classified in different endotypes according to their genomic information (55).

Until the current day, there is no molecular host biomarker panel available as a point of care for physicians to take an informed decision of a precise intervention based on the diagnosis of immune function or the ability to monitor the changes in the status of sepsis patients. Recent studies such as the INFECT study underline the gain in performances to predict patient outcome using three immune markers together rather than each one solely. Morris et al. showed that the use of neutrophil CD88, HLA-DR, and T_regs_ percentage in standardized flow cytometry was able to predict the occurrence of secondary infection in critically ill patients. Furthermore, the team was able to propose a cut-off to identify immune dysfunction from day 3 to 9 of ICU admission (82). Such study and signature discovery studies emphasize the importance of using a multiplex-based panel of markers. Current ongoing studies such as the REALISM study might shed the light on the impact of using immune functional assays in combination with immunosuppression biomarkers to stratify critically ill patients (83).

To finally put all the pieces together, an array of biomarkers is needed to tackle the heterogeneity of endotypes and identifying different host responses. Eventually, such biomarkers could help personalize treatment based on where a patient resides in the spectrum of inflammation, or whether specific organs are failing (84). In order to benefit from immunotherapy for instance, patients need to be stratified using a multiplexing technology of a biomarker-based panel to characterize the immune status of the patients. Therefore, a “bundle” of biomarkers combined with IFA could provide a robust tool to help achieve the desired stratification of high-risk patients such as the immunosuppressed profile, to map the altered pathways, and achieve a tailored management.

**Figure 2:**
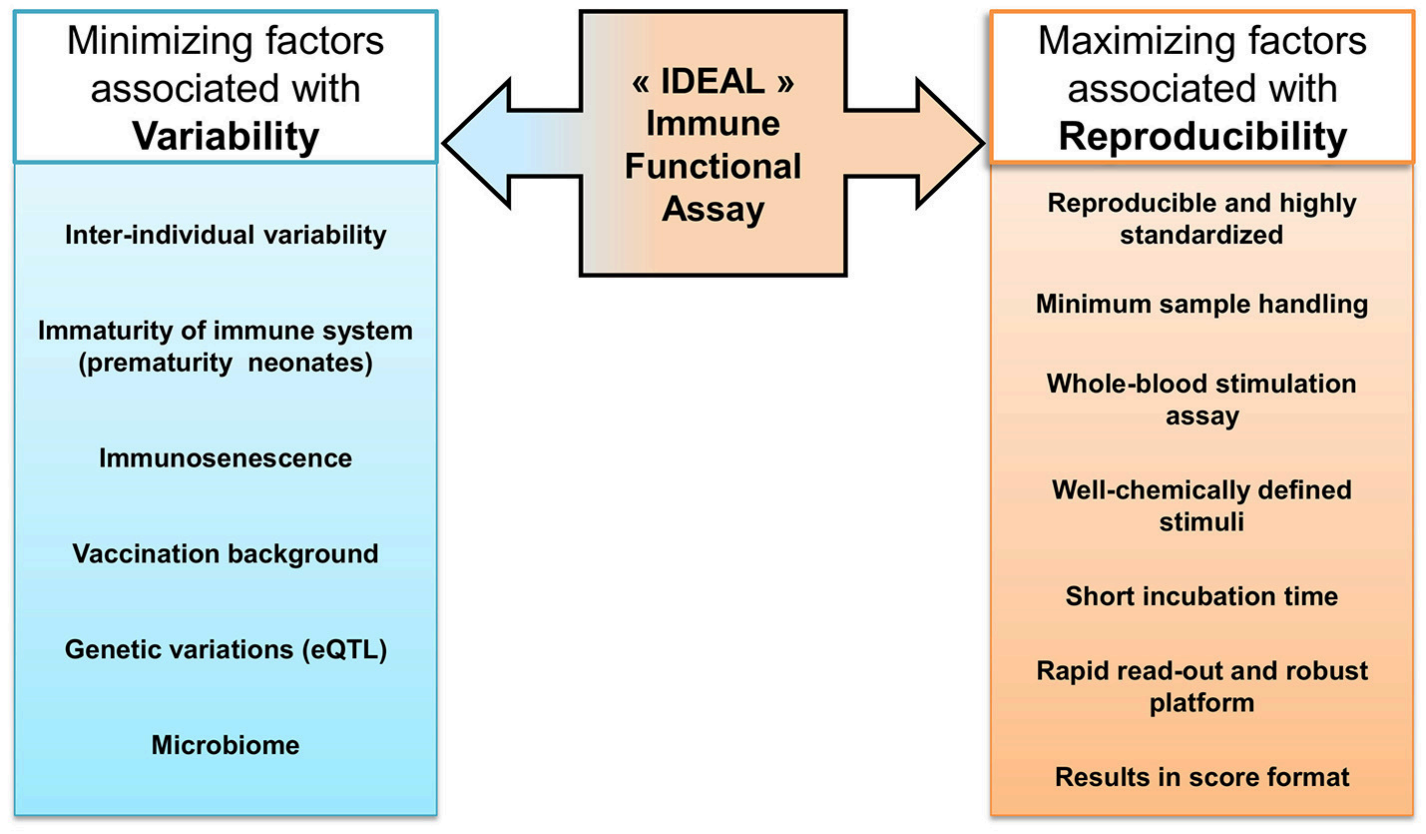
“Ideal” Immune Function Assay. Many intrinsic factors contribute to the increased variability among subjects, such as genetic factors or vaccination record. The ideal IFA should be minimally impacted by those factors that create diversity in the physiological context. Taking into consideration the challenge to interpret results due to inter-individual diversity, technical factors, as minimal sample handling or robust platforms, can be optimized to allow reproducibility between tests and decrease the bias accumulated during the manipulation workflow.

Research efforts are now directed toward precision medicine using several biomarkers to identify susceptible endotypes and predict the outcome of disease in order to intervene accurately. IFA paves the way to anticipate treatment responders from non-responders and functional measurement of the immune cells. Current immune function assays used in clinical routine for different applications have some limitations that hamper standardized reproducibility, however they are still used by default as being the better option. To develop an “ideal IFA,” as illustrated in Figure 2., the first requirement is to be highly standardized, reproducible across labs, demand a minimum of sample handling and require less technical skills. To accomplish this aim, the stimulant has to be consistent and a chemically well-defined molecule used worldwide with the same properties. Besides, the stimulation time has to be shortened as much as possible to be used at the bedside. For the evaluation of the response, the choice of the read-out and the technical platform is critical to obtain quick and accurate upshots. Ideally, the results should be processed within the day to get back to the patient in the least time. Results have to be obtained or smoothly transformed into a special format that can be promptly interpreted by clinicians, to be used in their evaluation and decision-making. Indeed, an acceptable result has to be able to map a patient's disorder into a specific category of treatment and/or management care. The future objective for IFA development is the procurement of the result in a “score” format. This resulting score has to reflect the accuracy and sensitivity of the test but above all can be easily interpreted by clinicians to guide in the decision making. Laboratory assay, preclinical development and clinical relevance are key steps to translate as a point of care. Interaction among multidisciplinary staff and opening channels of discussion are indispensable to improve the rigor of diagnostic performance to achieve precision in patient management. Although, the heterogeneity among subjects will always exist and will remain a challenge for the classification of endotype to be evaluated by an IFA test.

## Author contributions

Conception and structuring of the manuscript were done by JT, ST-A, and FM. Drafting the manuscript was done by CA-V, DT, and JT. Final revision and editing was done by all authors.

### Conflict of interest statement

CA-V, DT, FM, LV, and JT are employed by an *in-vitro* diagnostic company, bioMérieux. The views presented in this editorial are the personal opinion of the authors and do not necessarily represent the viewpoint, strategy, or opinions of bioMérieux. The remaining author declares that the research was conducted in the absence of any commercial or financial relationships that could be construed as a potential conflict of interest.
